# Inhibition of microRNA 128 promotes excitability of cultured cortical neuronal networks

**DOI:** 10.1101/gr.199828.115

**Published:** 2016-10

**Authors:** K. Melodi McSweeney, Ayal B. Gussow, Shelton S. Bradrick, Sarah A. Dugger, Sahar Gelfman, Quanli Wang, Slavé Petrovski, Wayne N. Frankel, Michael J. Boland, David B. Goldstein

**Affiliations:** 1 Institute for Genomic Medicine, Columbia University Medical Center, New York, New York 10032, USA;; 2 University Program in Genetics and Genomics, Duke University, Durham, North Carolina 27708, USA;; 3 Computational Biology and Bioinformatics, Duke University, Durham, North Carolina 27708, USA;; 4 Biochemistry and Molecular Biology, University of Texas Medical Branch, Galveston, Texas 77555, USA;; 5 Department of Genetics and Development, Columbia University Medical Center, New York, New York 10032, USA;; 6 Department of Medicine, The University of Melbourne, Austin Health and Royal Melbourne Hospital, Melbourne, Victoria 3052, Australia;; 7 Department of Neurology, Columbia University Medical Center, New York, New York 10032, USA

## Abstract

Cultured neuronal networks monitored with microelectrode arrays (MEAs) have been used widely to evaluate pharmaceutical compounds for potential neurotoxic effects. A newer application of MEAs has been in the development of in vitro models of neurological disease. Here, we directly evaluated the utility of MEAs to recapitulate in vivo phenotypes of mature microRNA-128 (miR-128) deficiency, which causes fatal seizures in mice. We show that inhibition of miR-128 results in significantly increased neuronal activity in cultured neuronal networks derived from primary mouse cortical neurons. These results support the utility of MEAs in developing in vitro models of neuroexcitability disorders, such as epilepsy, and further suggest that MEAs provide an effective tool for the rapid identification of microRNAs that promote seizures when dysregulated.

Epilepsy is a chronic disease that encompasses a broad spectrum of brain disorders characterized by recurrent and unprovoked seizures. It is the fourth most common neurological disorder, affecting people of all ages, and often includes additional debilitating neurological and cognitive consequences (Henshall 2014). While there has been substantial progress in identifying epilepsy genes (Epi4K Consortium and Epilepsy Phenome/Genome Project 2013), the underlying cause of disease remains unknown in most cases (Pandolfo 2011; Noebels 2015; Zhu et al. 2015). Some lines of evidence indicate that a subset of the genetic causes of epilepsy reside outside of protein coding genes, with microRNAs (miRNAs) being one area of recent attention (Henshall 2014; Zucchini et al. 2014; Noebels 2015; Wang et al. 2015).

miRNAs are 20- to 23-nucleotide (nt) single-stranded RNAs that modulate post-transcriptional gene expression through imperfect base-pairing with target messenger-RNAs. miRNAs are thought to be particularly important in the complex gene regulation programs that occur in the brain (Kuss and Chen 2008). In fact, a number of miRNAs are known to regulate neuronal processes and morphology, including the critical role of the miR-200 family in neuronal differentiation (Pandey et al. 2015) and the importance of finely tuned miR-134 expression in dendritic spine morphology (Schratt et al. 2006). Recent literature indicates a clear link between miRNAs and epileptogenesis. One of the most striking examples demonstrated that mice lacking *Mir128-2*, one of the two genes that encode mature miR-128, in dopaminergic neurons developed fatal seizures within 3 mo of age (Tan et al. 2013).

Here, we evaluated the utility of microelectrode array (MEA) technology to recapitulate in vivo excitability phenotypes due to inhibition of mature miR-128 in cultured cortical neuronal networks. We inhibited miR-128 expression in mouse cortical neurons by overexpressing a miRNA sponge, a competitive miRNA inhibitor, and recorded neuronal activity using an MEA, which captures the extracellular field potentials of electrically active cells. We identified significant increases in neuronal activity following miR-128 knockdown. Hence, in this study we describe an in vitro excitability phenotype due to miRNA regulation and establish a paradigm that can be used for effective identification of miRNAs that promote seizures when dysregulated.

## Results

### Verification of miR-128 knockdown

**Figure 1.**
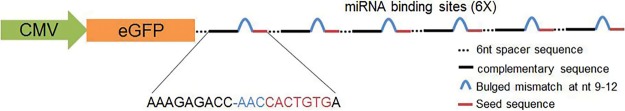
Sponge design in pLCE lentiviral transfer vector. The miR-128 sponge sequence is comprised of six tandem repeats of sequence complementary to miR-128. The sequence is designed with a mismatch at nucleotides 9–12 and each repeat is spaced by a 6- to 8-nt sequence. The sponge is driven by a CMV promoter downstream from a reporter *eGFP* gene. miRNA binding sequence is AAAGAGACCAACCACTGTGA.

**Figure 2.**
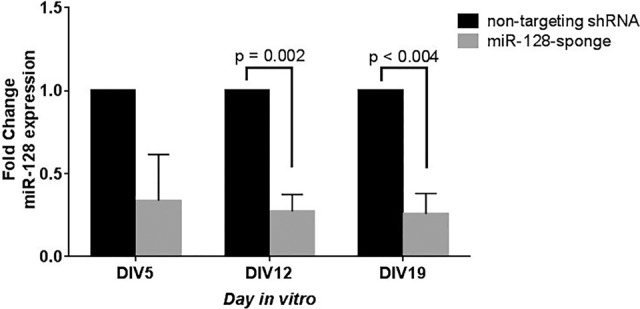
Verification of miR-128 knockdown via TaqMan assay. Inhibition of miR-128 results in a 67%, 73%, and 75% decrease of miR-128 expression in comparison to control on DIV5, DIV12, and DIV19, respectively. The control nontargeting shRNA is shown in black, and the miR-128 sponge is shown in gray. Data are represented as the mean of three biological replicates and three technical replicates each ± SEM (*n* = 3). *P*-value determined by multiple *t*-tests.

A miRNA sponge, delivered via lentiviral transduction, was used to inhibit miR-128 in primary dissociated cortical neurons from post-natal day 0 mice on the day of dissection (DIV0) (Fig. 1; Supplemental Table S1). To control for effects of lentiviral transduction, we tested two controls that expressed either a nontargeting shRNA or a sponge containing sequences complementary to an artificial miRNA (Ebert et al. 2007; Elcheva et al. 2009; Dylla and Jedlicka 2013). Each control behaved similarly in comparison with the miR-128 sponge (Supplemental Figs S3, S6). Approximately 15% of neurons were transduced using the miR-128 sponge (Supplemental Fig S4). There was no significant difference in cell viability between neuronal networks expressing a control sponge and networks expressing the miR-128 sponge (Supplemental Fig S5). Knockdown efficiency was evaluated using a TaqMan expression assay specific to mature miR-128. The engagement of miRNAs with their targets can induce degradation of the miRNA via tailing and trimming (Cazalla et al. 2010; Marcinowski et al. 2012; Rüegger and Großhans 2012). Inhibition of miR-128 with the sponge resulted in a 67%, 73%, and 75% decrease in expression on day five in vitro (DIV5), DIV12, and DIV19, respectively (Fig. 2). miR-128 targets several genes in upstream signaling events that activate MAPK3/1 by phosphorylation (Tan et al. 2013). We confirmed via immunoblot that sponge-mediated miR-128 knockdown leads to modest but consistent MAPK3/1 activation on DIV12 and DIV19 (Supplemental Fig. S7). The small effects on MAPK3/1 pathway activation are likely due in part to the low transduction efficiency achieved (15%) (Supplemental Fig. S4).

### miR-128 inhibition results in increased neuronal activity in cultured neuronal networks

**Figure 3.**
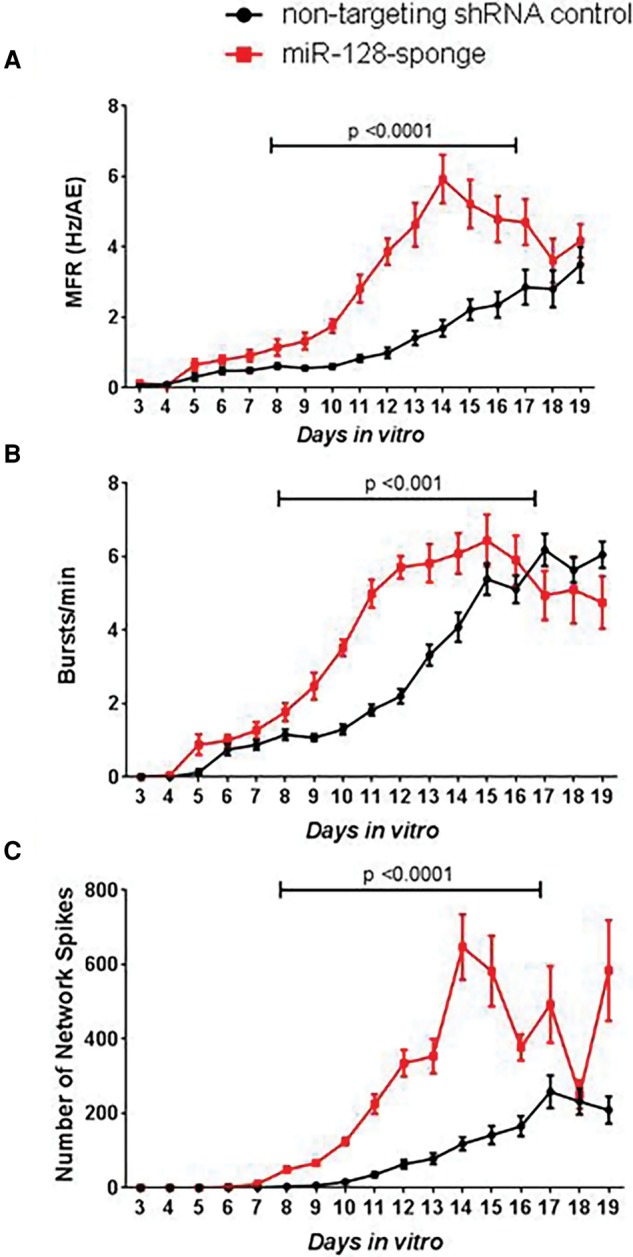
Inhibition of miR-128 increases neuronal activity. (*A*) MFR, (*B*) burst rate, and (*C*) number of network spikes of miR-128 knockdown cultures (red) are significantly increased in comparison to control cultures (black) between DIV8 and DIV16. The *x*-axis on all charts is DIV3 to DIV19. Data are represented as the mean of 17 wells transduced with the control nontargeting shRNA (*n* = 17) and 16 wells transduced with the miR-128 sponge (*n* = 16) ± SEM. *P*-values determined using permutation test (Supplemental Methods).

We evaluated neuronal activity patterns following miR-128 knockdown in vitro. Fifteen minutes of activity data from control neuronal networks and networks expressing the miR-128 sponge was collected by MEA every day starting on DIV3 (*n* = 3 independent MEA experiments, three to six biological replicates per experiment). Fifteen minutes of recording data captures more than 10,000 spikes per well; when data from three wells or more are available, this length of recording provides information sufficient to make statistically sound decisions about neuronal activity patterns. MEA data provide a metric for cultured neuronal network activity and synchronicity via measurement of changes in spike, burst, and network events. Spike and burst rates provide a metric for the overall activity of the neuronal network, with more spikes and bursts corresponding to higher activity. Network events include synchronous network spikes and bursts in which 16 or more electrodes (out of a total of 64) capture activity simultaneously. While our data focused on spikes, bursts, and network events, several additional activity features were extracted from the data, including the number of active electrodes, mean firing rate (MFR) normalized to the number of active electrodes, burst rate and duration, network spike and burst rate, and percentage of total spikes in bursts and network events across the cultured neuronal network (Fig. 3; Supplemental Table S2).

There was clear evidence of increased neuronal activity in the miR-128 knockdown neurons in comparison to control transduced cells. The MFR is considered to be an indicator of neuronal excitability and action potential frequency (Traub et al. 1996; McConnell et al. 2012). Transduction with the miR-128 sponge resulted in significantly increased MFR starting at DIV8 and persisting until DIV16 (permutation test, *P* < 0.0001) (Fig. 3A; Supplemental Fig. S1; Supplemental Methods). The effect of miR-128 knockdown on frequency of burst and synchronous network events was further investigated. The frequency of bursts and network events, including network level spikes and bursts, provides a readout of network level communication and activity. The miR-128 knockdown neurons showed a marked increase in the bursts per minute across active electrodes (permutation test, *P* ≤ 0.001) (Fig. 3B; Supplemental Fig. S1), number of synchronous network bursts per second (Mann-Whitney *U* [MW*U*] test and combined *P*-value by Fisher's combined probability test, *P* = 4.0 × 10^−7^) (Supplemental Table S2; Supplemental Methods), and number of network spikes (permutation test, *P* < 0.0001) (Fig. 3C; Supplemental Fig. S1). Together, these data demonstrate that miR-128 inhibition increases neuronal activity.

**Figure 4.**
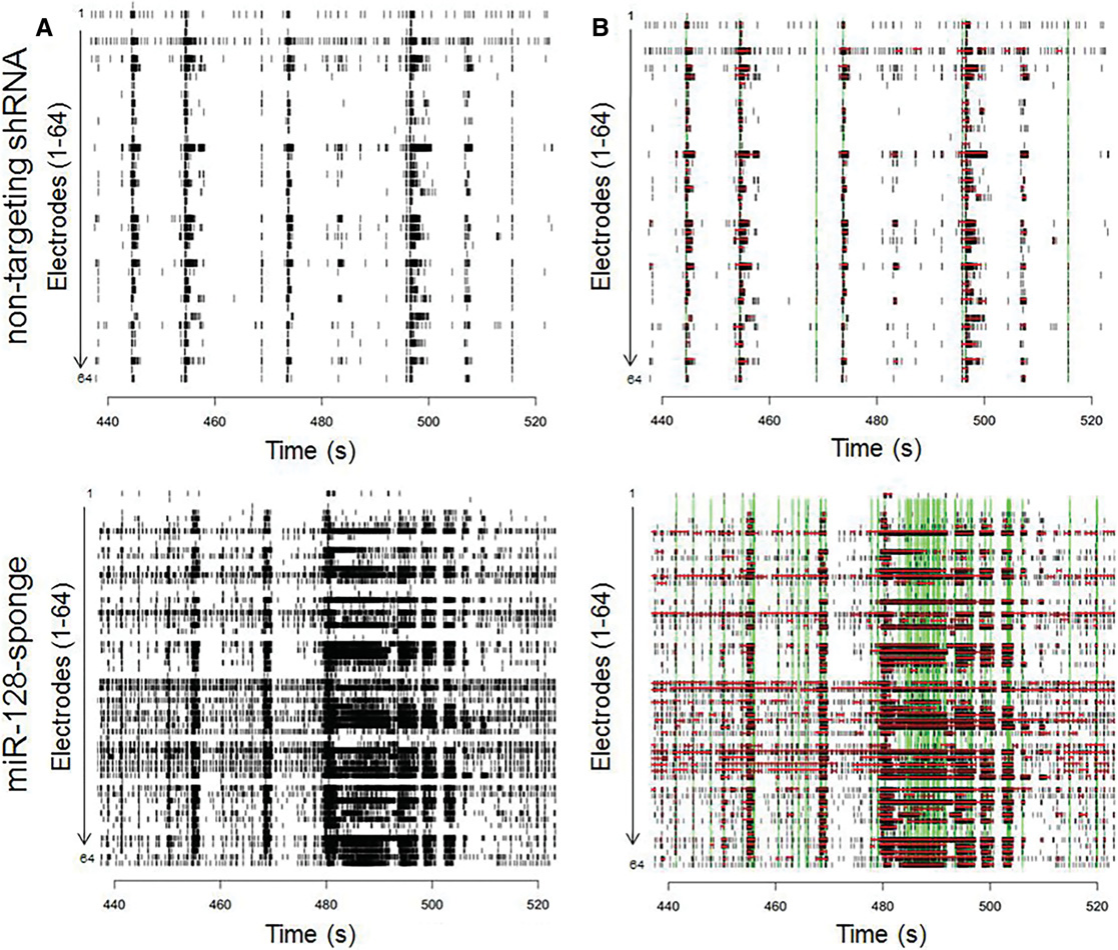
Raster plot illustrating network organization. (*A*) miR-128 knockdown results in short bursts with increased number of spikes per burst (*bottom* panel). (*B*) Increased burst activity is depicted with red horizontal bars and increased network events, including network spikes and network bursts, in green vertical bars. This raster plot depicts activity from 440–520 sec during the 15-min recording on DIV12 of a single representative control and miR-128 knockdown well.

Next, we examined whether miR-128 inhibition resulted in a change in the organization of bursts. miR-128 knockdown cultures showed decreased inter-burst intervals and increased duration of bursts (MW*U* test and combined *P*-value by Fisher's combined probability test, *P* = 1.5 × 10^−5^ and *P* = 6.3 × 10^−7^, respectively) (Supplemental Fig. S2) relative to controls. This indicates that miR-128 knockdown in cultured neuronal networks results in more numerous and longer lasting bursts, which occur in rapid succession with very short periods of inactivity between bursts (Fig. 4), representative of a change in organized burst patterns of the network. The burst patterns observed are directly correlated to the MFR and characteristic of hyperactive cultured neuronal networks.

We also evaluated activity-independent features. Activity-independent features are those that take into account the activity of the network by normalizing by the firing rate and include the percentage of spikes occurring in bursts and network events. The percentage of spikes within bursts is a metric of network activity organization, whereas percentage of spikes in network events is a feature of network synchronicity. The percentage of spikes in bursts significantly increased in knockdown cultures (Supplemental Table S2), indicating that single firing events in knockdown cultures lead to bursts more often than in control networks. There were no differences between knockdown and control groups in the percentage of spikes in network events (Supplemental Table S2). Together, these data indicate that miR-128 knockdown cultures are hyperactive but are not more synchronous, which is consistent with the in vivo neuronal excitability of *Mir128-2*–deficient mice (Tan et al. 2013). It should be noted that our transduction efficiency was only ∼15% across experiments (Supplemental Fig. S4), yet we observe striking and reproducible network phenotypes, thus highlighting the importance and sensitivity of neuronal networks to the expression levels of miR-128.

### miR-128 inhibition results in increased neuronal activity in mature cultured neuronal networks

To investigate whether the effect of miR-128 inhibition on cultured neuronal network properties is an acute effect on functioning cultured neuronal networks, rather than an effect of their development, we inhibited miR-128 expression in mature cultured neuronal networks (DIV19) to compare with DIV0 experiments described above. Significant increases in MFR and burst rate were noted between DIV28 and DIV36 in comparison to controls (permutation test, *P* = 0.0039 and *P* = 0.0293, respectively). Other activity-dependent features were elevated but did not reach statistical significance. Furthermore, there was no significant difference between the MFR of wells transduced with a nontargeting shRNA control and untransduced cells (Supplemental Fig. S8). Synchronicity patterns of network events remained unchanged between groups, as noted in DIV0 experiments. Together, these results show that inhibition of miR-128 in mature culture also results in an excitability phenotype and is not attributed to an effect on network development.

## Discussion

In this study, we inhibited miR-128 expression in primary dissociated cortical neurons and analyzed the resulting activity patterns of the neuronal networks that developed. The MFR observed for the control populations was within range of published values using MEA systems (Eytan and Marom 2006; Cao et al. 2012; Bateup et al. 2013; Colombi et al. 2013; Lignani et al. 2013). We found that inhibition of miR-128 resulted in increased MFR, increased burst rate, and an increase in the number of network events in both immature and mature cultures. Further, miR-128 inhibition led to a decrease of the inter-burst intervals and an increase in burst duration. Collectively, these features are thought to be hallmarks of excitability in cultured neuronal networks (Swartzwelder et al. 1987; Sombati and Delorenzo 1995; Bacci et al. 1999).

Thus, we have shown that MEAs can be used to detect the effect of genetic perturbations in cultured neuronal networks that reflect in vivo findings. Further, we have shown that the excitability effect due to inhibition of miR-128 is relevant to other neuronal populations in the brain outside of dopaminergic neurons (Tan et al. 2013). This highlights the utility of the cultured neuronal network–MEA paradigm for developing in vitro models of neurological disease. Our work complements a number of studies that successfully used cultured neuronal networks coupled with MEAs to investigate epilepsy-causing mutations (Lignani et al. 2013; Gullo et al. 2014) and neurotoxic effects (McConnell et al. 2012), together providing strong support for the use of MEAs to model neurological disorders. The results further demonstrate the utility of this paradigm to specifically investigate miRNAs.

The same methodology can be applied to the catalog of known miRNAs. A large number of miRNAs have been identified, the vast majority of which have not been assessed for epileptogenic or anti-epileptogenic potential. Profiling studies have revealed changes in the expression levels of over 100 miRNAs in experimental epilepsy, consisting of just under 20% of known brain-expressed miRNAs (Henshall 2014). Of these, 20 have been reported in more than one study, and only five have been assessed for epileptogenic potential in model systems: miR-128 (Tan et al. 2013), miR-34a (Sano et al. 2012), miR-132 (Huang et al. 2014), miR-134 (Jimenez-Mateos et al. 2012), and miR-184 (McKiernan et al. 2012). Our data provide motivations for using MEAs as a tool to systematically screen the list of implicated miRNAs, which will in turn allow researchers to prioritize findings and focus additional studies to those validated to have a neuronal effect.

Furthermore, identifying miRNAs that either promote or protect against seizures may provide novel directions for therapy. Drugs that target miRNAs offer the unique opportunity to target biological networks rather than individual components. Newly developed small molecules and drugs can be tested for efficacy of reverting in vitro seizure phenotypes related to miRNA dysregulation. We highlight the use of cultured neuronal networks coupled with MEAs as a paradigm for detecting effects of miRNA modulation on the generation of seizure-like activity in vitro.

## Methods

### Sponge design

We designed a stably expressing lentivirus sponge to inhibit miR-128 according to a previously described protocol (Ebert et al. 2007). The sequence comprised six tandem repeats partially complementary to the mature miRNA sequence. Each repeat possessed a perfectly complementary seed sequence (nucleotides 2–8 of miRNA sequence). Nucleotide 9 was not included, and nucleotides 10–12 were designed to mismatch with the miRNA sequence. This is called the bulge, which hinders the miRNA processing proteins from cleaving the sponge sequence. Nucleotides 13–21 were designed to be exactly complementary to the miRNA sequence. Each repeat was spaced by a 6- to 8-nt sequence. The sponge sequence was expressed in the pLenti-CMV-eGFP (pLCE) lentiviral transfer vector (Fig. 1; Supplemental Table S1). A nontargeting shRNA with a scrambled sequence was used as a control. Similar data were achieved using a control sponge based on the *Cxcr4* gene sequence (Supplemental Figs. S3, S6), which includes nine repeats of an artificial miRNA that is not complementary to any known miRNA and is also positioned downstream from the *eGFP* reporter gene in the pLCE vector.

### Neuronal culture

Primary cortical neurons were dissociated from the brains of post-natal day 0 (P0) C57BL/6J wild-type mice and plated onto 12-well MEA plates (Axion Biosystems). All procedures involving mice were approved by the Division of Laboratory Animal Resources at Duke University and the International Animal Care and Use Committee at Columbia University. One to seven days before dissection, each 12-well plate was treated with polyethyleneimine and then washed four times with distilled water and allowed to dry overnight in a sterilized hood. On the day of dissection, the wells were coated with laminin and incubated at 37°C during the dissection. Laminin was removed by aspiration prior to seeding dissociated neurons onto the wells. P0 pups were isolated and immediately decapitated. Dissociated neurons from the entire cerebral cortex were used and cultured in Neurobasal-A media supplemented with B27. Media was 50% changed on DIV3 and every other day thereafter. For additional methods, including cell culture, miR-128 expression analysis by qRT-PCR, and immunoblotting technique, please see the Supplemental Methods.

### MEA data analysis

Spontaneous network activity was recorded for 15 min every day using the Axion Biosystems Maestro MEA at 37°C. We collected raw data and a spike list file, which contains the spike data for each recording. These files were used downstream to extract spike, burst, and network data, using code adapted from sjemea package created by Stephen Eglen (Eglen et al. 2014). At the end of the experiment, activity data were inspected to remove inactive electrodes and wells. We determined the parameters for detecting neuronal bursts and network events based on published reports and experimentation (McConnell et al. 2012; Mack et al. 2014). In order for an electrode to be considered active, we required that at least five spikes per minute were recorded. Wells in which fewer than 16 electrodes were active for >30% of the days of recording were considered inactive and removed from analyses. For synchronous network events, at least 16 electrodes (25% of the total in a well) were required to participate in a network event in order for the network event to qualify as a network spike or burst. Events with less participating electrodes were filtered. By using the adapted code, activity variables were calculated (Supplemental Methods). To analyze data over time, the values for each well between DIV8 and DIV16 were combined and a MW*U* test was performed. The labels on each well were then shuffled and permuted 10,000 times to create 10,000 data sets that were tested for significance using a MW*U* test. The *P*-value of the actual data set was then compared to the distribution of the *P*-values from the permuted data sets to determine whether differences between the groups were significantly different from random. For additional information pertaining to MEA data analysis and statistical testing, please see the Supplemental Methods.

## Data access

The data from this study, including the data generated by the Axion Maestro system and the output of the data after running it through our software, as well as the analysis package (IGM.MEA_0.3.2.tar.gz) used to generate activity features discussed here, are publicly available on Figshare.com (https://figshare.com/projects/Inhibition_of_microRNA-128_promotes_excitability_of_ cultured_cortical_neuronal_networks/15200). The following individual data sets are within the database, followed by their digital object identifiers:

1. Spikelist files generated during activity recordings: https://dx.doi.org/10.6084/m9.figshare.3543728.v2, https://dx.doi.org/10.6084/m9.figshare.3521726.v4, https://dx.doi.org/10.6084/m9.figshare.3521621.v3, https://dx.doi.org/10.6084/m9.figshare.3521435.v5, https://dx.doi.org/10.6084/m9.figshare.3521291.v4.
2. The R code used to process the spikelist files: https://dx.doi.org/10.6084/m9.figshare.3543341.v2.
3. Data files generated from processing spikelist files: https://dx.doi.org/10.6084/m9.figshare.3543467.v1, https://dx.doi.org/10.6084/m9.figshare.3524531.v3, https://dx.doi.org/10.6084/m9.figshare.3524483.v1, https://dx.doi.org/10.6084/m9.figshare.3524447.v1, https://dx.doi.org/10.6084/m9.figshare.3524360.v2.

## Supplementary Material

Supplemental Material
