# Supplementary material for: Inhibition of microRNA 128 promotes excitability of cultured cortical neuronal networks
Source: Genome Res. 2016 Oct;26(10):1411–6. doi: 10.1101/gr.199828.115 (PMC5052052; doi:10.1101/gr.199828.115)
Supplement: Supplemental Material [file supp_26_10_1411__index.html]

Inhibition of microRNA 128 promotes excitability of cultured cortical neuronal networks — Supplemental Material 

# Inhibition of microRNA 128 promotes excitability of cultured cortical neuronal networks

## Supplemental Material

- Supplemental\_Material.pdf
