# Supplementary material for: Inhibition of microRNA 128 promotes excitability of cultured cortical neuronal networks
Source: Genome Res. 2016 Oct;26(10):1411–6. doi: 10.1101/gr.199828.115 (PMC5052052; doi:10.1101/gr.199828.115)
Supplement: Supplemental Material [file supp_gr.199828.115_Supplemental_Material.pdf]

Supplemental Materials

Table of Contents:

Supplemental Tables……………………………………………………………………………………pages 1-2

- Table 1: Sponge sequences
- Table 2: Changes in neuronal network activity due to miR-128 inhibition

Supplemental Figures:…………………………………………………………………………………pages 3-10

- Fig 1: Inhibition of miR-128 increases neuronal activity in comparison to a non-targeting shRNA
- Fig 2: Inhibition of miR-128 results in changes in neuronal activity organization
- Fig 3: Inhibition of miR-128 increases neuronal activity in comparison to a cxcr4-control-sponge
- Fig 4: Transduction of primary neuronal cultures with either miR-128-sponge or cxcr4-control-sponge result in similar transduction efficiencies
- Fig 5: Cell toxicity does not impact network activity
- Fig 6: Verification of miR-128 knockdown via qRT-PCR TaqMan assay
- Fig 7: Increased MAPK3/1 activation
- Fig 8: Inhibition of miR-128 in mature cultures results in an excitability phenotype

Supplemental Methods: ………………………………………………………………………………..……pages 11-14

- Sponge Design and Lentiviral Production
- Cortical Dissociation and Culture
- Verification of miR-128 Inhibition via TaqMan and Western Blot Analysis
- Cell viability
- MEA Data Analysis
  - Bursts Detection
  - Network Burst Detection
  - Fisher's combined probability test

Supplemental References…………………………………………………………………………..………page 15

Supplemental Table S1

Sponge sequences. Table showing the exact complimentary sequence to the mature microRNA and the miR-128-sponge sequence incorporating edits described in Methods and Supplemental Methods. The cxcr4-control-sponge sequence is also shown. Italicized letters indicate flanking restriction sites for cloning. Lowercase letters indicate spacer sequences. Uppercase letters indicate repeat sequences.

	Sequence
miR-128	UCACAGUGAACCGGUCUCUUU
reverse complement	AAAGAGACCGGTTCACTGTGA
miR-128-sponge	*ctcgag*AAAGAGACCAACCACTGTGAgaatatgaAAAGAGACCAACCACTGTGAagatatAAAGAGACCAACCACTGTGAttgaaaAAAGAGACCAACCACTGTGAgtaataAAAGAGACCAACCACTGTGAaagtatAAAGAGACCAACCACTGTGA*gaattc*
cxcr4-control-sponge	*ctcgagcacttctagt*AAGTTTTCAGAAAGCTAACAgttgAAGTTTTCAGAAAGCTAACAgttgAAGTTTTCAGAAAGCTAACAtctagtAAGTTTTCAGAAAGCTAACAgttgAAGTTTTCAGAAAGCTAACAgttgAAGTTTTCAGAAAGCTAACAtctagtAAGTTTTCAGAAAGCTAACAgttgAAGTTTTCAGAAAGCTAACAgttgAAGTTTTCAGAAAGCTAACA*tctagatttgaatt*

Supplemental Table S2

Changes in neuronal network activity due to miR-128 inhibition. Table showing the list of features investigated for each experiment using a non-targeting shRNA as a control. All features, except synchronicity, are direct or indirect indicators of cultured neuronal network excitability.

Feature	Measure of	Direction of change of miR-128 knockdown cells	Experiment 1 permutation test, p-value	Experiment 2 permutation test, p-value	Experiment 3 permutation test, p-value	Combined p-value, Fisher's combined probability test
Mean firing rate across active electrodes	Activity	Increase	<0.001	<0.001	<0.001	4.1 x 10-10
Total number of bursts	Activity	Increase	0.006	<0.001	0.011	1.3×10^{-6}
Bursts per minute	Activity	Increase	0.007	<0.001	0.005	7.0×10^{-7}
Percentage of spikes in bursts	Network organization	Increase	0.006	<0.001	0.003	4.1×10^{-7}
Burst duration	Network organization	Increase	0.001	0.003	0.001	6.3×10^{-7}
Inter-burst intervals	Network organization	Decrease	0.001	0.001	0.110	1.5×10^{-5}
Number of network spikes	Activity	Increase	<0.001	<0.001	0.006	1.8×10^{-8}
Number of network bursts per second	Activity	Increase	<0.001	<0.001	0.0018	4.0×10^{-7}
Number of spikes in network bursts	Activity	Increase	0.0084	<0.001	<0.001	1.6×10^{-6}
Percentage of spikes in network bursts	Synchronicity	Unchanged	0.089	0.154	0.167	0.059

Supplemental Fig S1

Inhibition of miR-128 increases neuronal activity in comparison to a non-targeting shRNA. Figure illustrating individual data from each experiment. Row 1 shows the mean firing rate across three experiments (y-axis = MFR/AE); row 2 shows the bursts per minute (y-axis = bursts/minute); row 3 shows the number of network spikes (y-axis = number of network spikes). x-axis = days *in vitro* (DIV). Experiment 1: control wells n = 6, miR-128-sponge transduced wells n = 5; Experiment 2: control wells n= 6, miR-128-sponge transduced wells n=6; Experiment 3: control non-targeting shRNA wells n = 5, miR-128-sponge transduced wells n=5.

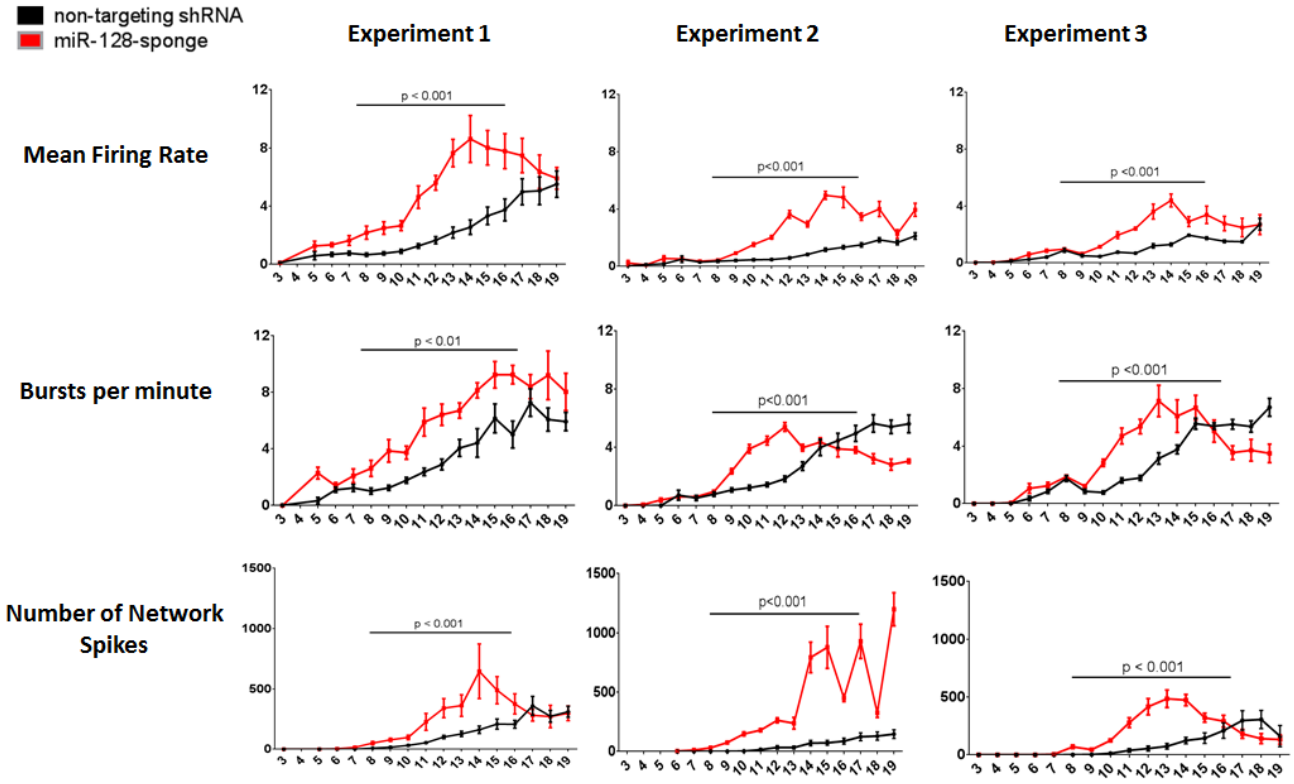

Supplemental Fig S2

Inhibition of miR-128 results in changes in neuronal activity organization. Row 1 shows cumulative distribution graphs of burst duration across three replicate experiments, Maximum Interval method. The duration of bursts is increased when miR-128 is inhibited. Row 2 shows cumulative distribution graphs of inter-burst intervals (IBI) across three replicate experiments, Maximum Interval method. IBI decreases when miR-128 is inhibited. x-axis = time (in seconds) x 10bins (ie: 50 = 5 seconds), y = axis = proportion. Black represents the control non-targeting shRNA virus and red represents the miR-128-sponge. Permutation p-values (see Supplemental Methods).

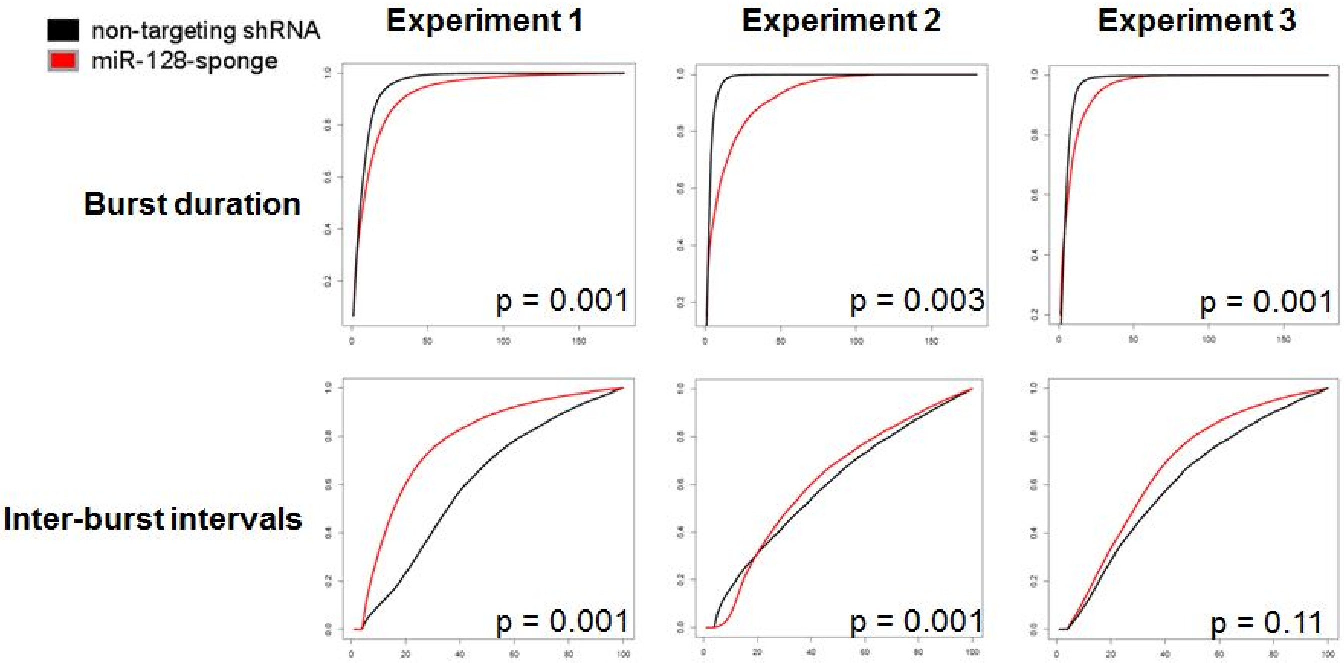

Supplemental Fig S3

Inhibition of miR-128 increases neuronal activity in comparison to a cxcr4-control-sponge. A) Sponge design of cxcr4-control-sponge in pLCE lentiviral transfer vector. B) Inhibition of miR-128 results in similar increases in neuronal activity when compared to a cxcr4-control-sponge as observed when compared to a non-targeting shRNA (Figure 2, Supplemental Fig 1). Row 1 shows the mean firing rate (y-axis = MFR/AE); row 2 shows the bursts per minute (y-axis = bursts/minute); row 3 shows the number of network spikes (y-axis = number of network spikes). x-axis = days *in vitro* (DIV). cxcr4-control-sponge wells (black) n = 3, miR-128-sponge transduced wells (red) n = 5. Permutation p-values (see Supplemental Methods).

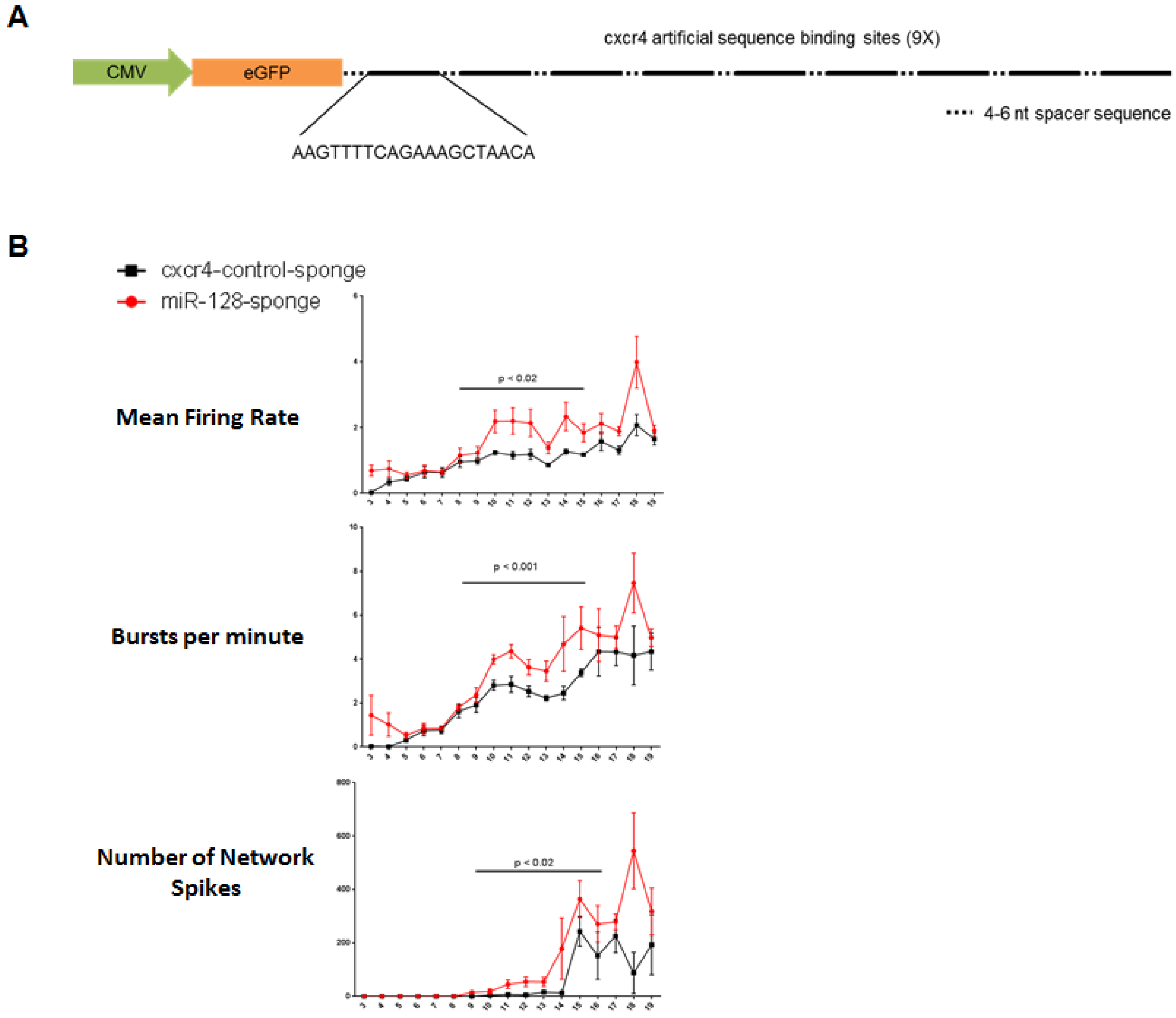

Supplemental Fig S4

Transduction of primary neuronal cultures with either miR-128-sponge or cxcr4-control-sponge results in similar transduction efficiencies. A) Figure showing representative images of transduction efficiency of the miR-128-sponge and a cxcr4-control-sponge on DIV5 after transduction. All images were taken at 20X on an EVOS FL cell imaging system. B) Quantification of transduction efficiency: The number of DAPI+ and GFP+ cells were counted using Image J from three independent 20X images of each condition. Number of GFP+ cells were normalized to total cell number (i.e. DAPI+). The average for miR-128-sponge transduced cells was 15.18 ± 0.8695. The average for cxcr4-control-sponge was 15.07 ± 0.1929. A MW*U* test determined that the groups were not significantly different (t-test, p = 0.7).

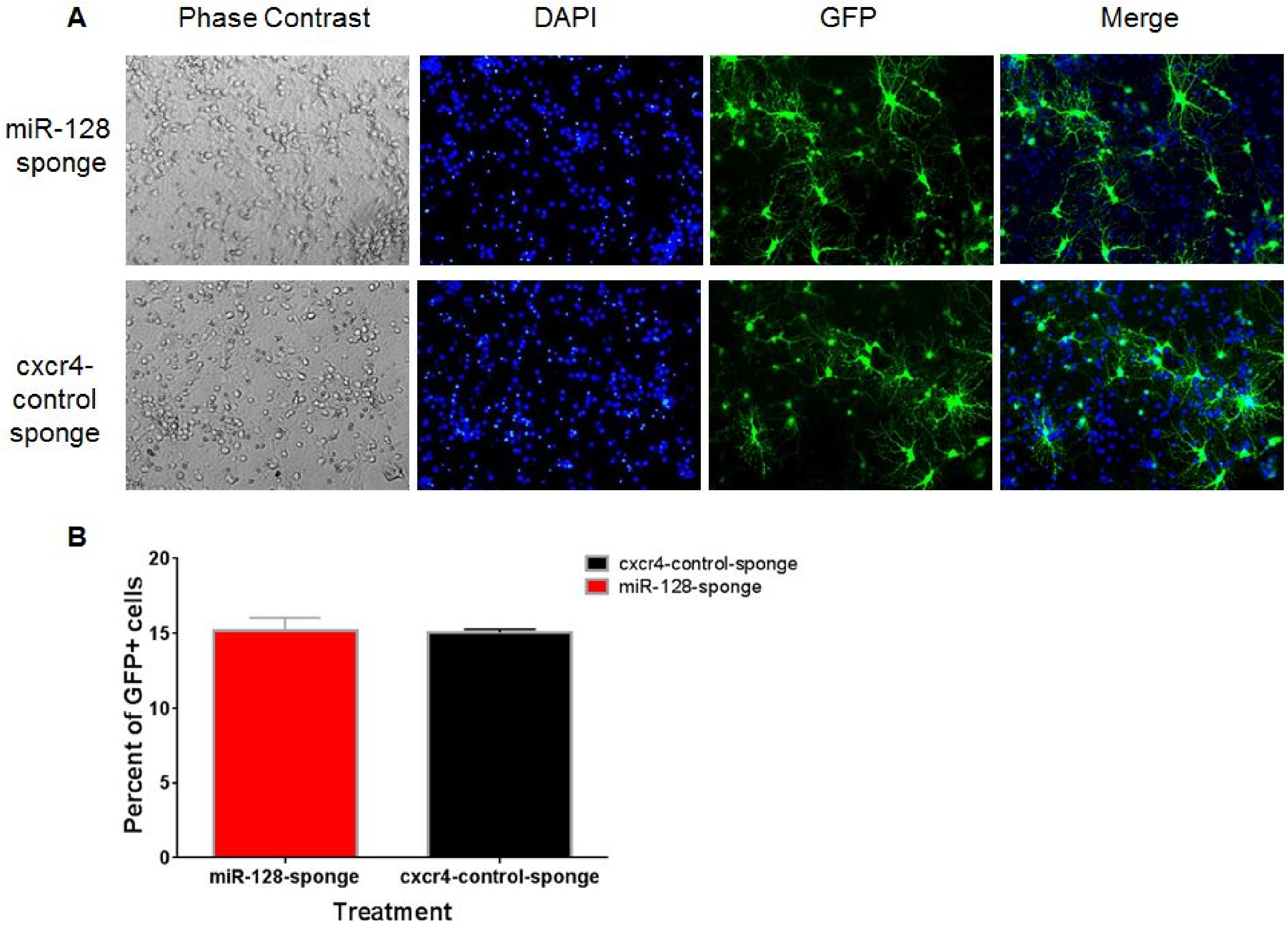

Supplemental Fig S5

Cell toxicity does not impact network activity. Neither the cxcr4-control-sponge nor the miR-128-sponge resulted in a significant difference in cell toxicity on DIV5 and DIV12 (determined by multiple t-tests). On DIV25 there was a 5% increase in cell toxicity in both groups ($p < 0.01$), but no difference between groups.

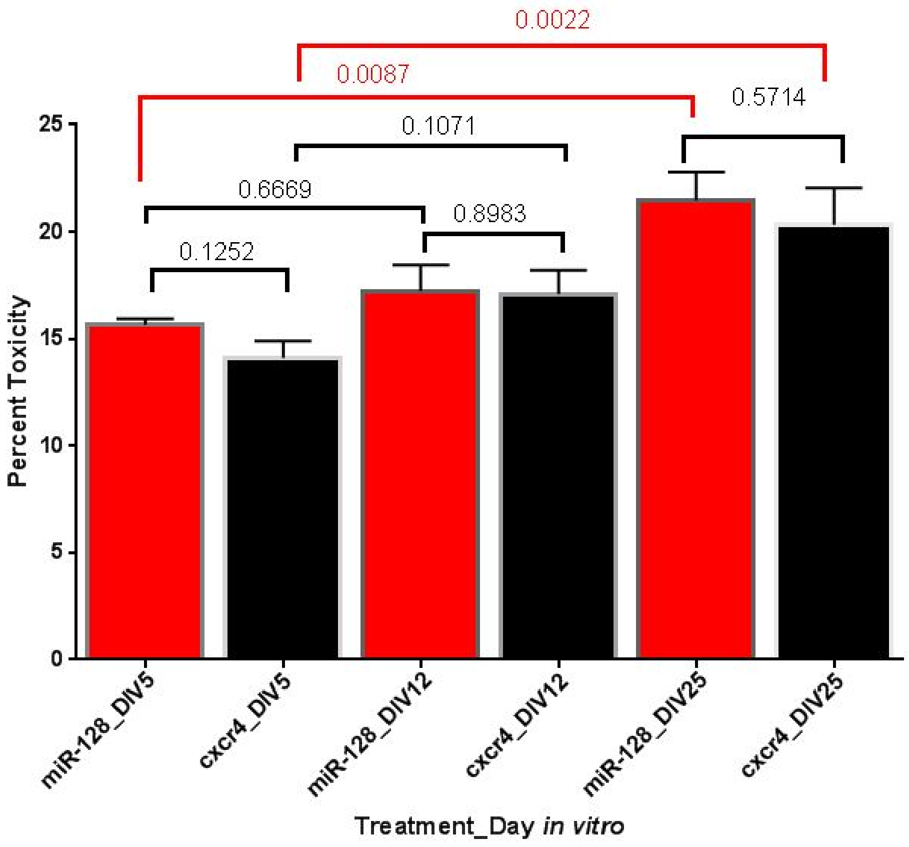

Supplemental Fig S6

Verification of miR-128 knockdown via qRT-PCR TaqMan assay. Inhibition of miR-128 results in similar decreases in expression on DIV5, 12, and 19 when compared to cxcr4-control-sponge (n = 3) as observed when compared to a non-targeting shRNA (n= 3, Figure 2)

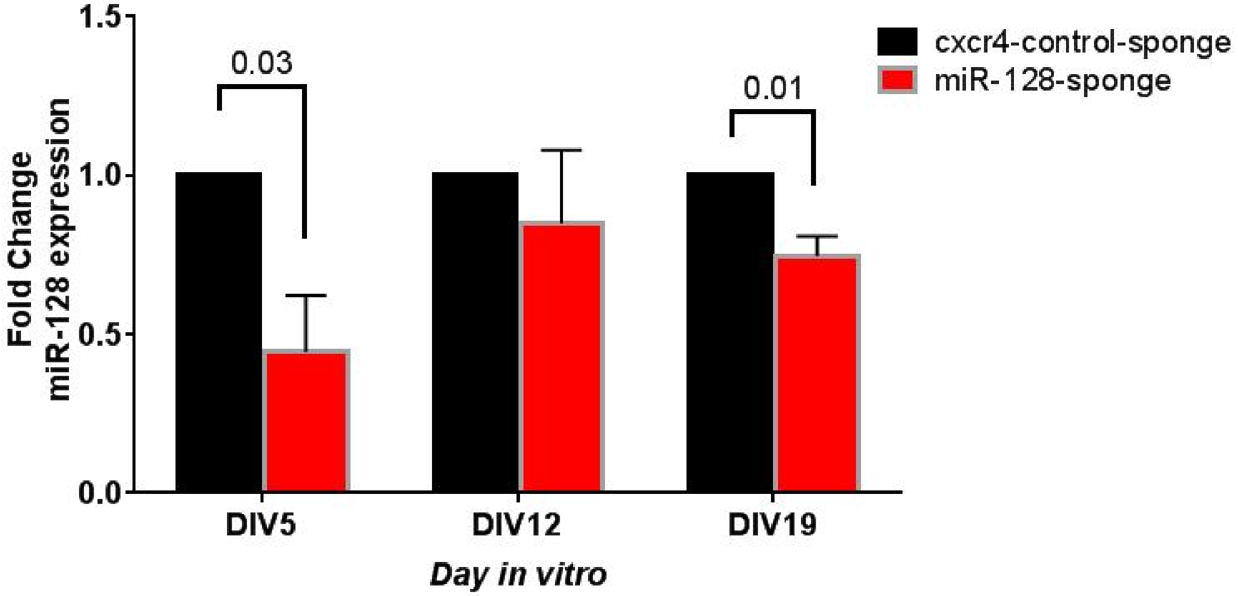

Supplemental Fig S7

Increased MAPK3/1 activation. Inhibition of miR-128 with a sponge results in a modest increase in MAPK3/1 activation on DIV12 and DIV19 when compared to the cxcr4-control-sponge. A) Representative western blot images of neuronal cell lysates on DIV5, DIV12, and DIV19. B) Quantification of replicate western blot densitometry determined by ImageJ shows a consistent but insignificant increase in MAPK3/1 phosphorylation (multiple t-tests). Data are represented as the mean of 3 replicates ± SEM. Each bar is shown relative to control (control = 1).

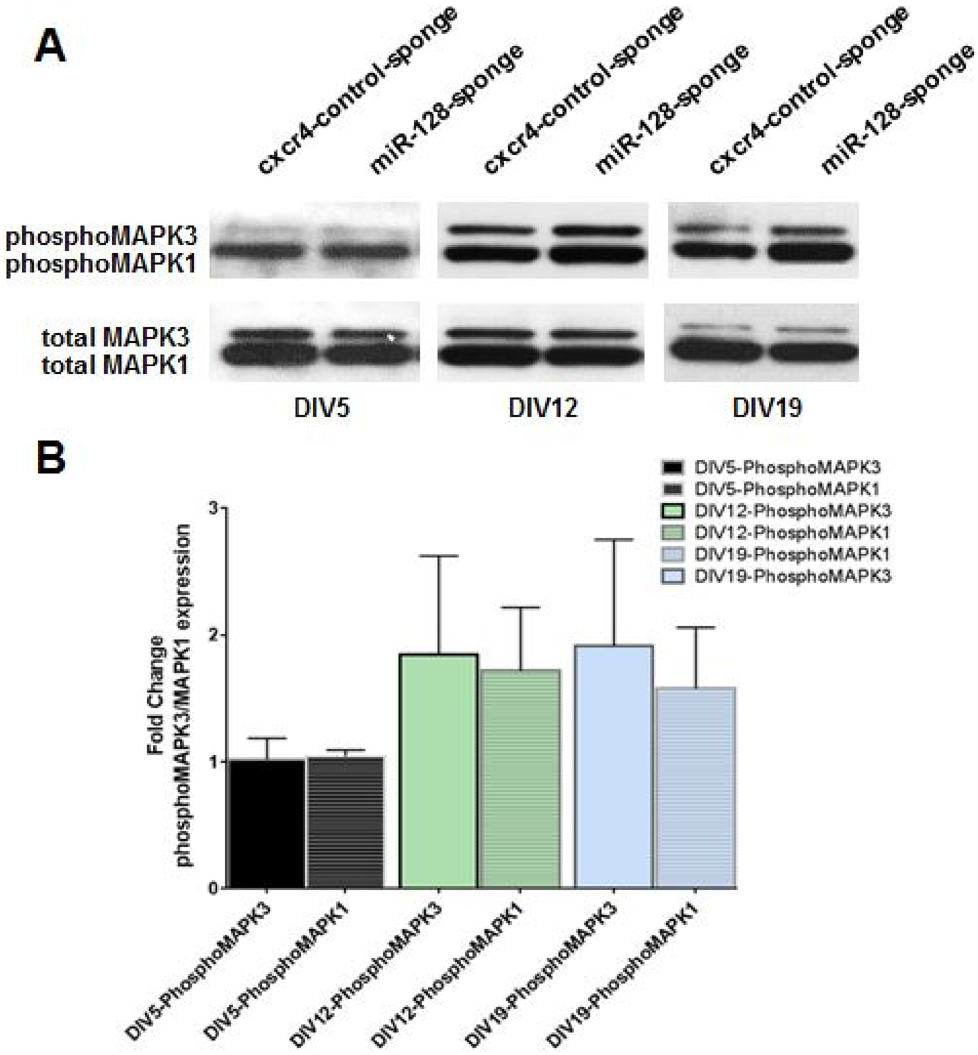

Comparison	p-value
DIV5-phosphoMAPK3 vs DIV12-phosphoMAPK3	0.7
DIV5-phosphoMAPK1 vs DIV12-phosphoMAPK1	0.4
DIV5-phosphoMAPK3 vs DIV19-phosphoMAPK3	0.7
DIV5-phosphoMAPK1 vs DIV19-phosphoMAPK1	0.9
DIV12-phosphoMAPK3 vs DIV19-phosphoMAPK3	0.9
DIV12-phosphoMAPK1 vs DIV19-phosphoMAPK1	0.9

Supplemental Fig S8

Inhibition of miR-128 in mature cultures results in an excitability phenotype. Mature cortical neuronal cultures were transduced with lentiviruses expressing either miR-128-sponge or non-targeting shRNA on DIV19 (arrow), MFR in miR-128 knockdown networks increased between DIV28-36 when compared to those expressing a control non-targeting shRNA (p=0.0039). There was no significant difference between shRNA and untransduced groups. Data from a 48-well MEA plate are represented as the mean of 16 wells ± SEM

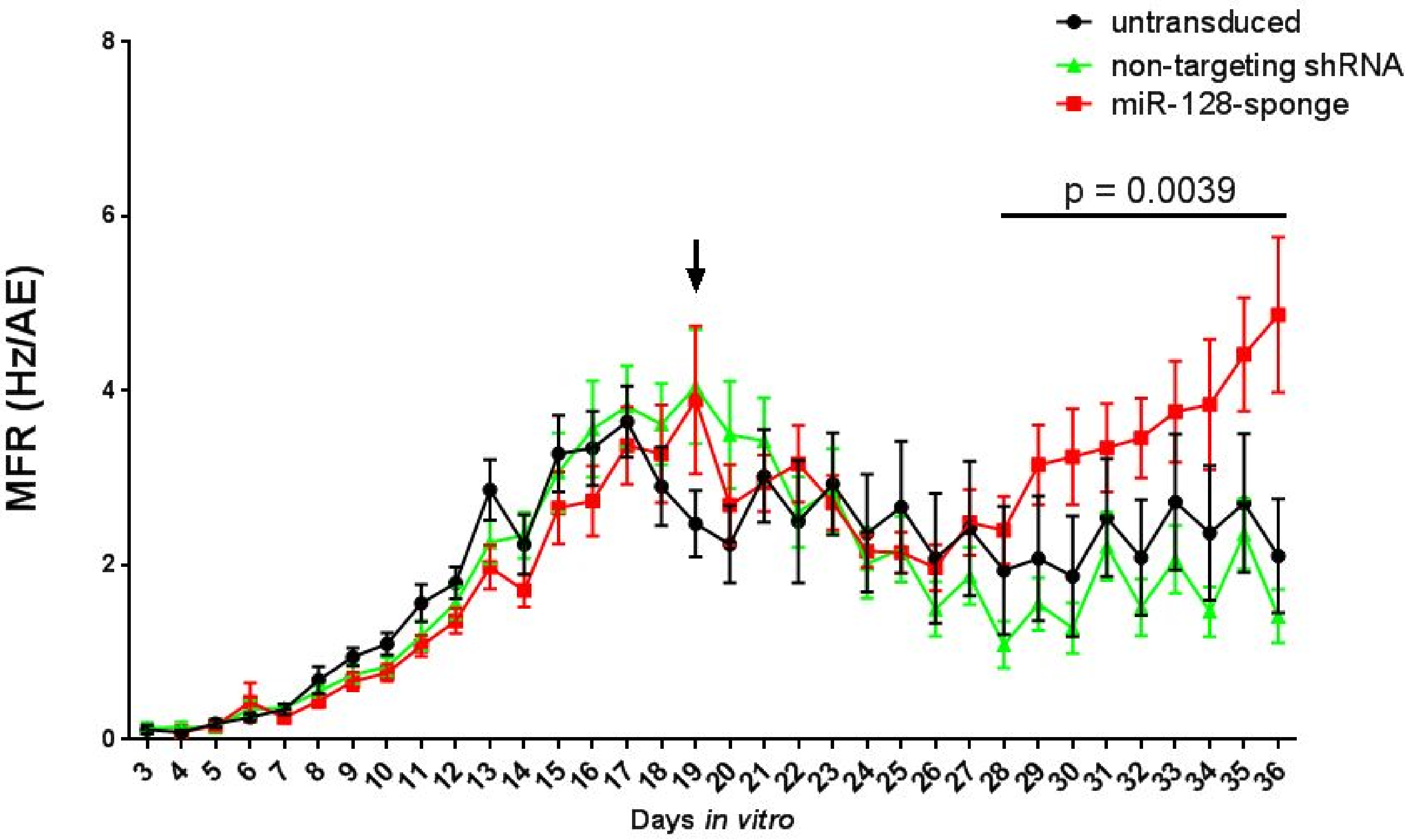

Supplemental Methods

Sponge Design and Lentiviral Production

We designed an artificial, stably expressing, lentivirus miRNA sponge according to the protocol described by (Ebert et al. 2007). The sponge sequence comprised of six tandem repeats partially complimentary to the miRNA sequence spaced by six nucleotide spacer sequences (Figure 1 and Supplementary Table 1). Each repeat possessed a perfectly complimentary seed sequence (nucleotide 2-8 of miRNA sequence). Nucleotide 9 was not included, and nucleotides 10-12 were designed to mismatch with the miRNA sequence. This is called the bulge, which hinders the miRNA processing proteins from cleaving the sponge sequence. Nucleotides 13-21 were designed to be exactly complementary to the miRNA sequence. The entire 152-nucleotide sequence was flanked on the 3' with an Xho1 restriction site and EcoR1 on the 5' and ordered from Integrated DNA Technologies (IDT) custom gene synthesis. The sponge sequence was cloned downstream of the eGFP reporter gene in the pLenti-CMV-eGFP (pLCE) lentiviral transfer vector using the Xho1 and EcoR1 restriction sites. Sponge lentiviral particles were generated by transfection of HEK293T cells with essential plasmids for lentiviral production using a modified protocol provided by Dr. Bryan Cullen's lab. Cells were cultured in Dubelcco's modified Eagle's media (DMEM) supplemented with 10% FBS and 1% Penicillin/Streptomycin at 37C in 5% CO2. Four million HEK293T cells were plated onto 10cm dishes 24 hours before transfection. Transfer sponge DNA, viral packaging vector pR8.75, and viral envelope vector pMD2G were diluted in 1ml serum free DMEM at a 17:17:7 ratio respectively and 1µg/µl PEI. After 15 minutes of incubation at room temperature, cells were transfected with the mixture. Media was changed 24 hours later to avoid PEI toxicity. Viral supernatant was harvested 48h and 72h after transfection, filtered through a 0.45 µm filter, aliquoted, and stored at -80C. The titer was determined by transducing HEK293T cells with serial dilutions of the virus in triplicate and counting the number of GFP positive foci 24 hours after transduction. The cxcr4-control-sponge was a gift from Dr. Bryan Cullen and was packaged similarly. The cxcr4-control-sponge contains nine repeats of an artificial miRNA (based on the *Cxcr4* gene sequence) that are spaced by four to six nucleotide spacer sequences. This control sponge has been used as a control for miRNA sponge research in several examples (Ebert et al. 2007; Dylla and Jedlicka 2013; Elcheva et al. 2009).

Cortical Dissociation and Culture

P0 C57BL/6J wild-type pups were isolated and immediately decapitated. The entire cerebral cortex was dissected and cut into small pieces under sterile conditions in cold Hank's Balanced Salt Solution (HBSS) buffer (Sigma Aldrich, H9394). All procedures involving mice were approved by the Division of Laboratory Animal Resources at Duke University and the International Animal Care and Use Committee and Institute for Comparative Medicine at Columbia University. The dissected cortex was then enzymatically digested using 0.25% trypsin (Life Technologies, 25200-056) for 8 minutes followed by 0.5ml deoxyribonuclease I from bovine pancreas (Sigma Aldrich, D4527) for 6 minutes at 37C. To neutralize the digestion, warm minimal essential media (MEM) (Life Technologies, 11140) was added (MEM + 10% FBS + 1% Penicillin/Streptomycin + 10mM Hepes Buffer + 3.0 g glucose) and the cells were centrifuged at 300RCF for 5 minutes. Cells were resuspended in warm MEM and separated by 8 trituration passes with a flame-polished glass Pasteur pipette. After two further centrifugations and trituration steps, cells were filtered through a 40µm cell strainer to remove debris. Three hundred thousand cells were seeded onto the 9mm conical well surface at the bottom of each well of the 12-well MEA plate and allowed to adhere for 1h at 37C in 5% CO_2. The appropriate volume of either the miR-128-sponge or the control virus (non-targeting shRNA or cxcr4-control-sponge; MOI5) was added to 1ml of warm MEM media and then added to each well for 2 hours. The media was then removed and replaced with 1ml warm Neurobasal-A (Life Technologies, 10888-022) + 1X B27 supplement (Life Technologies, 17504-044) + 1X GlutaMax (Life Technologies, 35050-061) + 1% Penicillin/Streptinomycin. Cultures were maintained at 37C in 5% CO2. Media was 50% changed every other day starting on DIV3. Glial growth was not chemically suppressed.

One to seven days before dissection each 12-well plate was coated with 0.05% - 0.1% polyethyleneimine for 45 minutes. The wells were then washed 4 times with distilled water and allowed to dry overnight in a sterilized

hood. On the day of dissection the wells were coated with 20ug/ml laminin and incubated at 37C during the dissection. Laminin was removed by aspiration prior to seeding dissociated neurons onto the wells.

Viral transduction on mature cultures was done on DIV19 on 48-well MEA plates. The 48-well plates were treated as described for 12-well plates, and 150,000 cells were seeded per well to account for decreased well surface area. The miR-128-sponge or control non-targeting shRNA was added to the well at MOI2.5. The media was then 50% changed on DIV20 and every other day thereafter.

Verification of miR-128 Inhibition via TaqMan and Western Blot Analysis

Seventy-five thousand dissociated cortical neurons were plated onto 96-well plates pre-coated with poly-D-lysine on the day of dissection and transduced with either miR-128-sponge or control lentivirus (non-targeting shRNA or cxcr4-control-sponge) as described for MEA plates. On DIV5, 12, and 19 cells were collected and total RNA was then extracted using Qiagen's RNeasy kit according to the manufacturer's guidelines (cat. # 74134). The TaqMan MicroRNA Reverse Transcription kit (Life Technologies, 4367038) was used to generate cDNA used in qRT-PCR. The hsa-miR-128 MicroRNA assay (Life Technologies, Assay ID: 002216) was used for detection of miR-128 expression. miR-128 expression was normalized to GAPDH expression and fold change was determined by comparing the miR-128-sponge transduced samples to control samples (non-targeting shRNA or cxcr4-control-sponge). p-values were determined using multiple t-tests.

On DIV5, 12, and 19 cells were lysed using RIPA buffer + 1X protease inhibitor cocktail (Sigma cat. # 88266) + 1X phosphatase inhibitor (PhospoStop, Roche Life Sciences). Equal amounts of protein were separated by SDS-PAGE, transferred to PVDF membranes (Millipore) and blotted using 1:1000 MAPK3/1 and phospho-MAPK3/1 primary antibodies (Cell Signaling cat. # 4348 and ThermoScientific, cat. # MA5-15173). Each primary antibody was dissolved in 5% BSA and probed overnight at 4C. Secondary antibodies were diluted in 5% BSA at 1:5000 for 1 hour at room temperature. Detection was achieved using Pierce Enhanced Chemiluminescence Western Blotting Substrate (ThermoScientific cat. # 32209). Image J densitometry was used to quantify the intensity of the MAPK3/1 and phosphoMAPK3/1 bands. The values for phosphoMAPK3 were divided by the values of totalMAPK3 and phosphoMAPK1 divided by totalMAPK1. The ratios of each were compared between the control and the experimental group to determine fold change.

Cell Viability

In order to determine the potential toxicity of the miR-128-sponge the CytoTox 96 Non-Radioactive Cytotoxicity Assay was used (Promega cat. # G1780). The assay is a colorometric assay that measures lactate dehydrogenase (LDH). LDH is released into the cell media upon cell lysis and therefore is used a measure of cell death. 50ul of cell media was collected from the wells of the MEA plate and added to a 96-well plate. 50ul of the cytotoxicity assay was then added to the 50ul media and incubated at room temperature for 30 minutes. The stop solution was added at the end of the incubation and the absorbance at 490nm was read in a plate reader. This assay was chosen as the method to detect cell viability because it allows for an indirect measure that does not perturb the cells on the MEA and therefore allows for subsequent MEA recordings without interference (Wallace et al. 2015).

MEA Data Analysis

Activity data was gathered every day for 15 minutes per day using Axion Biosystems Maestro microelectrode array at 37°C in a CO_2 gas controlled chamber. Each well on a 12-well plate is comprised of 64 electrodes on an 8 by 8 grid with each electrode capturing activity of nearby neurons. Each 48-well plate is comprised of 16 electrodes on a 4 by 4 grid. A Butterworth band-pass filter (200-3000HZ) and an adaptive threshold spike detector set at 7X the standard deviation of the noise were used during recordings. Raw data and a spike list files were recorded. Spike list files were used to extract additional spike, burst, and network features, using code adapted from sjemea package created by Stephen Eglen (Eglen et al. 2014). Activity data was inspected to remove inactive electrodes and wells. We required that at least 5 spikes/minute were recorded per electrode and discarded wells with fewer than 16 active electrodes (or 4 active electrodes in the case of the 48-well

plate) for more than 30% of total the recording days. We collected data on the number of active electrodes per well for every day of recording, and calculated how many wells had >15 active electrodes and divided by the total number of recording days. If a well had <16 electrodes active over the days of recording, it was filtered from additional analyses. We required that at least 16 electrodes (or 4 for the 48-well plate) participated in a spike or burst event to be considered a network event. Events with less participating electrodes were filtered. Using the adapted code, activity features were calculated.

To analyze data over time, a permutation test was performed in R. To generate each permutation dataset, the labels of each well (control vs experimental) were randomly shuffled 10,000 times, but data from each well were unchanged so as to preserve the correlations between the time points within wells while breaking relationships between the group (control vs experimental) and subsequent outcome. A MW*U* test was performed for each of the 10,000 data sets comparing distributions in outcome between groups and a p-value was recorded. A permutation p-value for the MW*U* test was computed as the proportion of permuted data MW*U* p-values that were less than or equal to the MW*U* p-value from the original un-permuted dataset.

Burst Detection

Bursts were detected using the Maximum Interval burst detection algorithm (Neuroexplorer software, Nex Technologies, Littleton, MA) implemented in the sjemea R package (Eglen et al. 2014). We required that a burst consists of at least 5 spikes and lasts at least 50 milliseconds and that the maximum duration between two spikes within a burst to be 0.05. Adjacent bursts were further merged if the duration between them is less than 0.8 second. These parameters were chosen based on the literature (Johnstone et al. 2010; Mack et al. 2014) and on in-house experimentation. The same parameters were used for all recordings.

Further examination of bursting patterns was done for each of the DIV recordings of approximately 900 seconds (15 minutes). We assessed the following bursting features: inter burst interval, inter spike interval within burst, number of spikes in a burst, burst duration and spike frequency within a burst. For each feature, we calculated a normalized histogram for each electrode with values between zero and one, to adjust for variability between different electrodes. To accommodate for electrodes with variable levels of activity, we averaged the data across all electrodes of each group (control or experimental). The normalized histograms were calculated for both groups by averaging all the electrodes belonging to a group. Then, distributions for each of the five bursting features were derived from the control and experimental recordings. We tested for differences between the two distributions by calculating the Earth Mover's Distance (EMD) from the R emdist package (Urbanek and Rubner 2012). Distance between distributions was then tested using a permutation test to evaluate the strength of the difference between the original distributions and 10,000 random sets. For each of 10,000 permuted datasets we computed an average of the normalized histograms within each group. Distributions were then compared for each of the burst features using both Earth Mover's Distance (EMD) and maximum distance (MD). Similar values (EMD and MD) were also computed for the original (unpermuted) data. A permutation p-value for EMD was computed as the proportion of permuted data EMD values that are equal to or greater than the original EMD value from the unpermuted dataset. A permutation p-value for MD was defined similarly.

To test several DIVs from the same experiment, the time window from DIV8-16 was selected for analyses. The DIV8-18 window was selected based on the development of the network. DIV8 was the first instance of synchronous network events detected in MEA experiments and was therefore chosen for the first day of analysis. DIV16 was chosen as the last day analyzed based on the return of the miR-128 excitability phenotype to normal levels in all experiments. Next, feature distributions of all participating electrodes in all selected DIVs were then combined to one dataset per group, on which the EMD and permutation tests were performed. The above method allows the observation of differences between groups along full recordings, while still accounting for variability of the activity between channels and wells that could arise from the different types of neurons in a cortical culture or the distance of a recorded neuron from an electrode.

Network Burst Detection

Network burst patterns at the well level were investigated using an in-house method that takes into consideration bursts at the electrode level as well as the synchronization of bursts across a well. Firstly, spike time within spike trains from all electrodes was normalized through binning. Since the spike detection algorithm sets a default minimum distance between any two spikes to greater than 2.16ms, we used a bin size of 2ms to guarantee that at most one spike is called within each bin. Secondly, a Gaussian Filter with a given window size (5 separate window sizes were used) was applied to smooth the binned spike train from each electrode. The smoothed signal was then further standardized to have a maximum signal value of 1. All smoothed signals at the electrode level were then further added together and smoothed again using the same Gaussian filter. The final result from this step is a smoothed signal at a given window size that measures the overall synchronization of all electrodes in a well, with larger values indicating higher level of synchronized bursting activities. Thirdly, the Otsu global thresholding method (Otsu 1979) is applied to the well level signal from the previous step to automatically detect the burst intervals. We chose this method for its simplicity and parameter free nature, although other methods, such as adaptive thresholding, can be utilized. Finally, based on the network burst intervals from Otsu thresholding, we collected all burst features at the well level, such as network burst frequencies, inter network burst intervals etc.

Fisher's combined probability test

In order to combine p-values from independent biological replicate experiments, Fisher's combined probability test was used. R scripts were developed to allow the input of multiple p-values to generate a combined p-value by the Fisher's combined probability test previously described in the literature (Fisher 1925; Kugler et al. 2010).

Supplemental References

Dylla L, Jedlicka P. 2013. Growth-Promoting Role of the miR-106a~363 Cluster in Ewing Sarcoma. *PLOS ONE* **8**: e63032.

Ebert MS, Neilson JR, Sharp PA. 2007. MicroRNA sponges: competitive inhibitors of small RNAs in mammalian cells. *Nat Methods* **4**: 721–726.

Eglen SJ, Weeks M, Jessop M, Simonotto J, Jackson T, Sernagor E. 2014. A data repository and analysis framework for spontaneous neural activity recordings in developing retina. *GigaScience* **3**: 3.

Elcheva I, Goswami S, Noubissi FK, Spiegelman VS. 2009. CRD-BP protects the coding region of βTrCP1 mRNA from miR-183-mediated degradation. *Mol Cell* **35**: 240–246.

Fisher R. 1925. *Statistical methods for research workers: Biological monographs and manuals.* Edinburgh,: Oliver and Boyd.

Johnstone AFM, Gross GW, Weiss DG, Schroeder OH-U, Gramowski A, Shafer TJ. 2010. Microelectrode arrays: A physiologically based neurotoxicity testing platform for the 21st century. *NeuroToxicology* **31**: 331–350.

Kugler KG, Mueller LA, Graber A. 2010. MADAM - An open source meta-analysis toolbox for R and Bioconductor. *Source Code Biol Med* **5**: 3.

Mack CM, Lin BJ, Turner JD, Johnstone AFM, Burgoon LD, Shafer TJ. 2014. Burst and principal components analyses of MEA data for 16 chemicals describe at least three effects classes. *NeuroToxicology* **40**: 75–85.

Otsu N. 1979. A Threshold Selection Method from Gray-Level Histograms. *IEEE Trans Syst Man Cybern* **9**: 62–66.

Urbanek S, Rubner Y. 2012. *emdist: Earth Mover's Distance*. https://cran.r-project.org/web/packages/emdist/index.html (Accessed August 5, 2015).

Wallace K, Strickland JD, Valdivia P, Mundy WR, Shafer TJ. 2015. A multiplexed assay for determination of neurotoxicant effects on spontaneous network activity and viability from microelectrode arrays. *NeuroToxicology* **49**: 79–85.
